# Supplementary material for: Complexome profiling of the Chlamydomonas psb28 mutant reveals TEF5 as an early PSII assembly factor
Source: Plant Cell. 2025 Mar 17;37(6):koaf055. doi: 10.1093/plcell/koaf055 (PMC12164590; doi:10.1093/plcell/koaf055)
Supplement: koaf055_Supplementary_Data [file koaf055_supplementary_data.zip › psb28_interactive.zip!/plots/intensity/qualityPlots/peptides/CYN28_Cre12.g561000.t1.1.html]

### Cre12.g561000.t1.1

Trivial name: CYN28  
SeqCoverage [median]: 23.10  
Mass: 35.51 kDa  
  
 

| Sequence | MissedCleavage | Mass [Da] | wtIntensity | muIntensity | Score |
| --- | --- | --- | --- | --- | --- |
| ARPEYRPVEFLITTGPGPVPR | 2 | 2351.28 | 1287.23 | 951.13 | 43.33 |
| LGAVEVPPTVQSNPEVSEASAFK | 0 | 2355.20 | 0.00 | 461.51 | 29.12 |
| SIIPEAEPVGR | 0 | 1166.63 | 0.00 | 1404.49 | 55.55 |
| VLAGEYIQAGK | 0 | 1147.62 | 840.48 | 0.00 | 50.04 |
| VVIGLYGNAVPR | 0 | 1256.72 | 429.86 | 3539.97 | 84.48 |
|  |  |  | 2557.6 | 6357.1 |  |
